# Protocol: systematic review and meta-analyses of birth outcomes for women who intend at the onset of labour to give birth at home compared to women of low obstetrical risk who intend to give birth in hospital

**DOI:** 10.1186/2046-4053-3-55

**Published:** 2014-05-29

**Authors:** Eileen K Hutton, Angela Reitsma, Julia Thorpe, Ginny Brunton, Karyn Kaufman

**Affiliations:** 1 Department of Obstetrics and Gynecology, Faculty of Health Sciences, McMaster University, Hamilton, ON, Canada; 2 Department of Midwifery Science, EMGO Institute for Health and Care Research, VU University Medical Center and the VU University, Amsterdam, The Netherlands; 3 Midwifery Education Program, Faculty of Health Sciences, McMaster University, Hamilton, ON, Canada; 4 EPPI-Centre, Social Science Research Unit, Institute of Education, University of London, London, UK

**Keywords:** Home childbirth, Infant mortality, Pregnancy outcomes, Midwifery care, Systematic review

## Abstract

**Background:**

There has been a renewed interest in the place of birth, including intended home birth, for low risk women. In the absence of adequately-sized randomised controlled trials, a recent Cochrane review recommended that a systematic review and meta-analysis, including observational studies, be undertaken to inform this topic. The objective of this review is to determine if women intending at the onset of labour to give birth at home are more or less likely to experience a foetal or neonatal loss compared to a cohort of women who are comparable to the home birth cohort on the absence of risk factors but who intend to give birth in a hospital setting.

**Methods:**

We will search using Embase, MEDLINE, CINAHL, AMED and the Cochrane Library to find studies published since 1990 that compare foetal, neonatal and maternal outcomes for women who intended at the onset of labour to give birth at home to a comparison cohort of low risk women who intended at the onset of labour to give birth in hospital. We will obtain pooled estimates of effect using Review Manager. Because of the likelihood of differences in outcomes in settings where home birth is integrated into the health care system, we will stratify our results according to jurisdictions that have a health care system that integrates home birth and those where home birth is provided outside the usual health care system. Since parity is known to be associated with birth outcomes, only studies that take parity into account will be included in the meta-analyses. We will provide results by parity to the extent possible.

**Systematic Review Registration:**

This protocol was registered with PROSPERO at http://www.crd.york.ac.uk/Prospero/ (Registration number: CRD42013004046).

## Background

Today intended birth at home in most well-resourced countries, outside of The Netherlands, accounts for up to only 3% of all births. There are extremes of response and support for women who plan a home birth. Some settings have infrastructure in place to support home birth while in other settings home birth is undertaken in a less supportive or even hostile environment [1,2]. Other variations across settings include differences in the training, skill level and scope of practice of home birth care providers, the criteria used to determine eligibility for home birth, and the ability for home birth practitioners to make timely referrals of women to specialist care or to transfer women into hospitals when needed. When women plan to give birth at home in a setting where this choice is largely unaccepted, they may encounter barriers in accessing specialised care should it be required after labour has started, which may have a direct influence on the birth outcome. The varying responses of obstetrical societies to intended home birth provide evidence of contrasting views. In the United Kingdom, the Royal College of Obstetricians and Gynaecologists guidelines are supportive of birth at home for low risk women, stating: “There is no reason why home birth should not be offered to women at low risk of complications and it may confer considerable benefits for them and their families” [3]. The current guideline of the American Congress of Obstetricians and Gynecologists states: “Women inquiring about planned home birth should be informed of its risks and benefits based on recent evidence. Specifically, they should be informed that although the absolute risk may be low, planned home birth is associated with a twofold to threefold increased risk of neonatal death when compared with planned hospital birth” [1]. This guideline is informed by a meta-analysis published in 2010 [4] that has been shown to be fundamentally flawed at many levels including errors in the analysis of the data [5,6]. Given the controversial research findings and opposing opinions, home birth studies continue to be conducted and published in various international locales. A large cohort study from the United Kingdom has been published reporting on outcomes for all women intending home birth as part of the National Health Trusts [7]. A Cochrane review of randomised controlled trials addressing this topic included one small trial, and noted that in the absence of adequately-sized randomised controlled trials on the topic of planned home compared to planned hospital birth, a systematic review and meta-analysis including observational studies should be undertaken [8].

Thus, the objective of our systematic review and meta-analyses is to determine if women intending at the onset of labour to give birth at home are more or less likely to experience a foetal or neonatal loss compared to a cohort of similarly low risk women who intend at the onset of labour to give birth in hospital.

We are aware that there are several ways of approaching this research objective, and plan to carefully analyse the research design of each study to ensure that a meta-analysis is only undertaken when the research questions of the included studies are similar. First, information about the study setting taken from the publication, correspondence with authors, and other published literature will inform us about the integration of home birth into the health care system. Studies from locations where home birth is considered well-integrated into the health care system will be analysed separately from studies of places where home birth is a less-acceptable option and not integrated into the health care system. Second, we will consider the criteria used to assemble the intended home birth and intended hospital birth cohorts in each study. One method is to include all intended home births in a given time frame regardless of their eligibility or obstetric risk status and to compare them to a group intending hospital birth and deemed to be at low obstetrical risk. This method reflects a pragmatic approach and will determine the safety of home birth in actual practice. The research question that will be answered using studies taking this approach is:

1. Do women who intend at the onset of labour to give birth at home experience a higher or lower incidence of foetal or neonatal loss compared to women at low obstetric risk who intend at the onset of labour to give birth in hospital?

A different method of assembling the study cohorts is to include only women who meet local eligibility criteria for home birth regardless of intended place of birth. The result of such a study provides information about place of birth under ideal circumstances where women’s choices align with professional recommendations. The research question that will be answered using studies taking this approach is:

2. Do women who intend to give birth at home and who meet their local eligibility criteria for home birth at the onset of labour experience a higher or lower incidence of foetal or neonatal loss compared to women who would have been eligible for home birth but intend at the onset of labour to give birth in hospital?

We will address both research questions to increase the applicability of our findings. Furthermore, because we are interested in outcomes within parity groups (nulliparous and multiparous) we will report findings for these sub-groups.

## Methods

### Eligibility criteria

To meet the objectives of this review, we will apply the following inclusion/exclusion criteria to studies of home birth:

1. The study must have a comparison group of women who intended to birth in hospital, and the women in this “intended hospital birth group” must be at similarly low risk for birth complications, as defined by the authors, as the women in the intended home birth group. In pragmatic study designs, the intended hospital birth group may in fact have been at a lower obstetrical risk than the intended home birth group.

2. The cohorts must be defined by the intended location of birth rather than the actual location of birth. Intrapartum transfers from the intended home to hospital should be captured in the intended home birth group, and inadvertent home births occurring in the intended hospital group should be captured in the hospital birth group. Both scenarios, for different reasons, have the potential to increase the risk of poor outcomes, and these outcomes should be analysed according to the intended place of birth.

3. We will exclude studies where the intention for a home birth is determined earlier in the pregnancy and not reconfirmed at the onset of labour. When complications of pregnancy arise prior to the start of labour, most women will rightfully plan to give birth in hospital despite previous hopes for a home birth.

4. The study must account for parity since outcomes are known to be different for nulliparous women compared to primiparous and multiparous women [9].

5. The study must provide some assurance that the cohort of those intending home birth is complete (no missing cases) within the jurisdiction, in order to eliminate selection bias. Furthermore, the study should indicate if any of the women in the home birth cohort fell outside of the eligibility criteria for home birth in the studied jurisdiction, essentially increasing the risk of complications for that cohort. The authors should indicate how they approached the inclusion or exclusion of these cases in the analyses.

6. The study must be published in a peer reviewed journal during or after 1990. There has been a steady decline in perinatal and neonatal mortality, and an increasing rate of Caesarean section and epidural use in the last 20 years [10]. The study should reflect today’s standards of obstetric care.

Studies with significantly incomplete outcome data (>10%) will be excluded from the meta-analyses.

### Information sources

We will search five electronic databases separately for relevant articles: Embase, Medline, and AMED using the OVID interface; CINAHL using the EBSCOhost interface, and the Cochrane Library. Reference lists from included articles and in particular from any systematic reviews will also be cross-checked.

### Search strategy

Our search strategy will use a combination of subject headings, keywords and free-text terms related to the specific intervention, such as (home birth; homebirth; home delivery; home childbirth). Because we anticipate that there may be a variety of study designs that present home birth outcomes, we will not include terms for methodology, as is recommended for reviews of non-randomised studies [11]. Each search will be limited to include only studies published after 1989 (see Additional file 1). We will not limit the search by language. In the event that we find studies that are not written in English, we will use translation services as needed.

### Study selection

Two reviewers will independently screen the titles and abstracts for full-text retrieval. A full-text screening form has been created, outlining the eligibility criteria for inclusion in the review (see Additional file 2). Two independent reviewers will assess eligibility, and any disagreements will be noted, and then discussed until consensus is reached. If consensus cannot be achieved, a third reviewer will be involved. Tables of included and excluded studies will be maintained.

### Studies with more than one comparison group

We anticipate that some studies will have more than one comparison group, for example, women intending home birth compared to those intending hospital birth under the care of midwives and to those intending hospital birth under the care of family physicians or those under the care of obstetricians. If we encounter this situation we will provide descriptive data for all of the groups (either home or hospital) that are included in each study, likely using table format. For the meta-analyses, we will attempt to combine the outcomes for the hospital groups, provided that the women in the groups being combined meet the eligibility criteria. Although this approach increases the potential of increasing variance of outcomes among the comparison groups, it also diminishes any potential for selection bias in making the determination of which group(s) to include [12]. If the data for some or all outcomes cannot be combined, we will choose the comparison group most likely to minimise confounders; that is for the study under consideration we will choose the comparison cohort where the women are most like women choosing home birth, and the care providers are most like those providing care at home within the study under review [12]. We will not include births planned to take place in birthing centers or other out-of-hospital institutions as a comparison group in the meta-analyses as these settings may face some of the same issues of access to emergency care that are encountered in the home setting.

### Data collection

Two reviewers will independently extract data from each of the included studies using a detailed data abstraction form (see Additional file 3). Data will include study information (population studied, year(s) of births reported on, and date and journal of publication), information about the population studied (age, gestational age, descriptors of socioeconomic status and parity) and study methods (design, methods of controlling for parity and other confounders, outcomes of interest, intervention and comparison group inclusion and exclusion criteria). The primary outcome will be derived using available event occurrences and when such derivation is not possible we will attempt to contact the author of the original paper to determine if data are available. We will record the source of data for each study and indicate the completeness of the data set. We will maintain a list of criteria by which the birth cohorts were found to be at low obstetric risk and how authors dealt with parity, which is known to be associated with both birth outcomes [9] and intended place of birth [13].

The degree of support for home birth and home birth care providers within the health care system is hypothesised to act as an effect modifier of the relationship between intended place of birth and birth outcomes [1,2]. We have termed this context for home birth as an ‘integrated’ versus ‘non-integrated’ home birth environment. In order to determine whether home birth and home birth providers are integrated into the health care system we will collect information from each study about whether practitioners were recognised care providers within the health care system and could facilitate smooth transition from home to hospital and transfer of care to consultants when needed. Where information in the article is not explicit, we will look to secondary sources for supporting evidence, and will consider information about recognition of midwifery, hospital admitting privileges for midwives or other home birth attendants, the presence of a statement regarding home birth from the jurisdiction’s society/association of obstetricians and how home birth is funded to help understand the context of the setting in which home birth is occurring. In addition, we will contact all authors of the included publications and ask them to complete a brief questionnaire (see Additional file 4), which will provide information about the degree of integration of home birth within their health care system at the time that the data were collected. We will make every attempt to ensure that we receive responses from authors for our questionnaire. We will report on which authors provided a response and which did not. In the event that an author does not reply, we will rely on other sources of information and cite them accordingly.

We will compare the likelihood of foetal or neonatal death, neonatal outcomes and maternal outcomes occurring after labour has begun and obstetric intervention between those who intended to give birth at home at the onset of labour and those who intended to give birth in hospital. Our primary outcome will be any foetal or neonatal death reported in the study. We anticipate that studies may report stillbirth rates, perinatal mortality, neonatal mortality or some combination of these. Because mortality will be reported in the same way within each study, we will include any reported death so that all studies can contribute to the primary outcome. We will report stillbirth rates, foetal mortality and neonatal mortality separately where possible. Additional neonatal outcomes will include admission to neonatal intensive care units (NICU), neonatal resuscitation (as defined in each study), and Apgar scores of less than seven at five minutes. Definitions (for example: mortality, resuscitation) used by the authors will be collected and included in a table that describes each included study. Apgar scores are assigned to neonates at one minute and five minutes after birth, and are meant to serve as a measure of the newborn’s transition to neonatal life. An Apgar score of less than seven at five minutes of age may indicate a problem, so we plan to collect the number of cases that fall below this cut point. Where it is available, we plan to record two components of neonatal resuscitation: the use of positive pressure ventilation and the use of chest compressions.

Data on maternal outcomes will include maternal death, postpartum hemorrhage, perineal trauma and infection. Postpartum hemorrhage has varying definitions; we will use the number of cases with estimated blood loss greater than 1,000 ml [14]. When these data are not available, we will record blood loss as defined by the authors and make note of any variation in definitions of hemorrhage. In addition, information about how blood loss was estimated will be collected, when available. Perineal trauma is defined in terms of degree of laceration, from first to fourth degree. A third or fourth degree laceration is significant in terms of morbidity so these data (and not first and second degree) will be collected. Data on infection will be abstracted along with the author’s definition of infection.

Data on obstetric interventions will be recorded, including the use of oxytocin for augmentation of labour, the use of epidural anaesthesia/analgesia for pain control, episiotomy, assisted vaginal delivery (vacuum or forceps) and Caesarean section. In studies where outcomes are presented as part of a composite, we will make every effort to obtain the data for the relevant components contributing to the composite. Transfer rates will be collected, including from home to hospital and from one hospital to another (or to another department). When available we will collect information about the timing of transfers and whether or not they were emergencies.

### Risk of bias in individual studies

Two reviewers will assess risk of bias for each of the included observational studies using the Newcastle Ottawa scale adapted for our study purposes [15]. For randomised controlled trials we will use the Cochrane risk of bias assessment tool, adapted to provide a risk of bias score [16]. For each included study, we will present the score for each item of the scale. Agreement between reviewers will be calculated for the study quality measures using Kappa.

### Summary measures

Our primary outcome is any reported foetal or neonatal mortality as a proportion of all births. We anticipate that studies may have used varying definitions of mortality, with differing time periods or exclusion criteria based on malformations. It is unhelpful to exclude studies (and potentially important outcomes) because different definitions were used. In addition to reporting any mortality, we will report on outcomes using a more traditional definition of time of death, such as perinatal death. We will report the crude mortality rate as well as the rate excluding malformed infants.

The association of intended place of birth with our primary outcome will be estimated using an odds ratio and 95% confidence interval. This approach will be used for all other outcomes measured as proportions. Odds ratios will be used because it is anticipated that at least some of the data for this review will be collected retrospectively, and some of the outcomes may occur relatively commonly, a situation where the odds ratio is the preferred measure [17].

### Synthesis of results

Data abstracted from studies will be entered into the Review Manager program. When individual case data are not available, effect estimates and their standard errors will be entered using the random effects inverse variance statistical method. Forest plots will be created for the outcomes of interest. Measures of consistency (I^2^) will be reported along with confidence intervals. The magnitude and strength of I^2^ will be interpreted according to the Cochrane Handbook [18]. If, for any outcome, a considerable amount of statistical heterogeneity exists between studies, data will not be pooled [18]. If a significant amount of heterogeneity is found between studies, the potential cause of heterogeneity will be explored by conducting subgroup analyses, stratifying by study design, type of hospital comparison group, country, varying home birth practices and baseline characteristics, as well as outcome definitions used by authors [18]. Where appropriate, quantitative pooling of results will use the Mantel-Haenszel statistical method and the random effects model to achieve a pooled odds ratio and 95% confidence interval.

### Risk of bias across studies

An inverted funnel plot will be created for the outcome of any perinatal or neonatal mortality to assess the risk of publication bias.

### Additional analyses

We will provide descriptive analyses of variation among study settings, and plan if possible, to stratify our meta-analyses based on those settings where home birth is an integrated service within the health care system and those where it is less integrated. Our *a priori* hypothesis is that studies reporting on births that take place in a health care system where home birth is well integrated as part of the health care system will have different (better) outcomes than in jurisdictions where home birth is not integrated and supported within the health care system. We will describe variations in defining low risk and in eligibility for home birth.

If it is possible, we will report outcomes stratified by parity (zero and greater than or equal to one).

### Reporting

We will report our findings in accordance with the Preferred Reporting Items for Systematic Reviews and Meta-Analyses (PRISMA) guidelines [19].

## Discussion

We will conduct a systematic review and meta-analysis of studies on births intended at the onset of labour to take place at home compared to hospital, taking into account factors such as parity and integration of home birth into the health care system. Our proposed systematic review and meta-analysis will contribute to the gap in the literature that has been identified by a recent Cochrane review [8] and will provide much needed information to both care providers and women and families planning for their births.

## Competing interests

The authors declare that they have no competing interests.

## Authors’ contributions

EKH and AR led the study design and drafting of the manuscript. JT, GB and KK contributed to the design of the protocol and manuscript revisions. All authors read and approved the final manuscript.

## Supplementary Material

Additional file 1 **“Search Strategy”.** Description: This file indicates the search strategy that will be used to identify studies that are potentially eligible for inclusion in our review. Click here for file

Additional file 2 **“Study Eligibility Form”.** Description: This form will be used by two independent reviewers to indicate whether or not each study meets the list of inclusion and exclusion criteria and therefore to determine eligibility for inclusion in our review. Click here for file

Additional file 3 **“Data Abstraction Form”.** Description: This form will be used by two independent reviewers to collect information from each included study, including counts and effect estimates for all outcomes of interest that were reported on. Additional sources may be used to complete parts of the form, such as the description of the study setting. Click here for file

Additional file 4 **“Questionnaire for Authors of Included Studies”.** Description: This file includes a questionnaire to be sent along with an accompanying cover letter to authors of all included studies to ascertain information about the integration of home birth into the health care system in the region at the time that each study was conducted. Click here for file
