# Supplementary material for: Protocol: systematic review and meta-analyses of birth outcomes for women who intend at the onset of labour to give birth at home compared to women of low obstetrical risk who intend to give birth in hospital
Source: Syst Rev. 2014 May 29;3:55. doi: 10.1186/2046-4053-3-55 (PMC4046441; doi:10.1186/2046-4053-3-55)
Supplement: Additional file 1 — “Search Strategy”. Description: This file indicates the search strategy that will be used to identify studies that are potentially eligible for inclusion in our review. [file 2046-4053-3-55-S1.doc]

**Search Strategy**

EMBASE

Limit: Publication date 1990 to current

|  | **Search** | **Search Field** |
| --- | --- | --- |
| 1. | home delivery | subject heading |
| 2. | home birth | keyword |
| 3. | home childbirth | keyword |
| 4. | homebirth | keyword |
| 5. | 1. or 2. or 3. or 4. |  |

AMED

Limit: Publication date 1990 to current

|  | **Search** | **Search Field** |
| --- | --- | --- |
| 1. | home delivery | keyword |
| 2. | home birth | keyword |
| 3. | home childbirth | keyword |
| 4. | homebirth | keyword |
| 5. | 1. or 2. or 3. or 4. |  |

Ovid MEDLINE®

Limit: Publication date 1990 to current

|  | **Search** | **Search Field** |
| --- | --- | --- |
| 1. | home childbirth | subject heading |
| 2. | homebirth | keyword |
| 3. | home delivery | keyword |
| 4. | home birth | keyword |
| 5. | 1. or 2. or 3. or 4. |  |

CINAHL

Limits: Publication date 1990 to current; Exclude Medline records; Research Article

|  | **Search** | **Search Field** |
| --- | --- | --- |
| 1. | home childbirth | MH Exact subject heading |
| 2. | home | TI Title |
| 3. | home | AB Abstract |

Cochrane Central Register of Controlled Trials

Limit: Registration date1990 to current.

|  | **Search** | **Search Field** |
| --- | --- | --- |
|  | Home childbirth | Record title |
| OR | Homebirth | Record title |
| OR | Home delivery | Record title |
