# Supplementary material for: Protocol: systematic review and meta-analyses of birth outcomes for women who intend at the onset of labour to give birth at home compared to women of low obstetrical risk who intend to give birth in hospital
Source: Syst Rev. 2014 May 29;3:55. doi: 10.1186/2046-4053-3-55 (PMC4046441; doi:10.1186/2046-4053-3-55)
Supplement: Additional file 2 — “Study Eligibility Form”. Description: This form will be used by two independent reviewers to indicate whether or not each study meets the list of inclusion and exclusion criteria and therefore to determine eligibility for inclusion in our review. [file 2046-4053-3-55-S2.doc]

**Study Eligibility Form**

| **Reviewer** | #1 AR | #2 JT | #3 EH |
| --- | --- | --- | --- |

| **Article ID** | RefWorks # | Author: | Year: |
| --- | --- | --- | --- |
|  | Journal: | | |

| **Population** | | |
| --- | --- | --- |
|  |  |  |
| Obstetrical risk status AND/OR home birth eligibility criteria of women included in the study is described | **YES** | **NO** |
| Parity was accounted for | **YES** | **NO** |

| **Intervention** | | |
| --- | --- | --- |
| Home birth planned at the onset of labour | **YES** | **NO** |
| Planned home birth group represents all home births during the study period OR describes cases removed | **YES** | **NO** |

| **Comparison** | | |
| --- | --- | --- |
| Hospital birth | **YES** | **NO** |
| Women at low obstetrical risk (as determined by authors) OR women met the local eligibility criteria for home birth | **YES** | **NO** |

| **Outcomes** | | |
| --- | --- | --- |
| Any of the following outcomes reported: | **YES** | **NO** |
| - Perinatal mortality |  |  |
| - Neonatal outcomes |  |  |
| - Maternal outcomes |  |  |
| - Obstetric Interventions |  |  |

| **Type of study** | | |
| --- | --- | --- |
| Intention-to-treat analysis (groups based on planned place of birth at onset of labour) | **YES** | **NO** |

| **Study inclusion** | |
| --- | --- |
| - All answers **YES** | **INCLUDE** |
| - Any answers **NO** | **EXCLUDE** |
| - Any answers uncertain | **Needs consensus** |
