# Supplementary material for: Protocol: systematic review and meta-analyses of birth outcomes for women who intend at the onset of labour to give birth at home compared to women of low obstetrical risk who intend to give birth in hospital
Source: Syst Rev. 2014 May 29;3:55. doi: 10.1186/2046-4053-3-55 (PMC4046441; doi:10.1186/2046-4053-3-55)
Supplement: Additional file 3 — “Data Abstraction Form”. Description: This form will be used by two independent reviewers to collect information from each included study, including counts and effect estimates for all outcomes of interest that were reported on. Additional sources may be used to complete parts of the form, such as the description of the study setting. [file 2046-4053-3-55-S3.doc]

**Data Abstraction Form**

| **Part A: Reviewer** | #1 AR | #2 JT |
| --- | --- | --- |

| **Part B: Article ID** | RefWorks # | Author: | Year: |
| --- | --- | --- | --- |
|  | Journal: | | |

| **Part C: Confirmation of Eligibility** | | | |
| --- | --- | --- | --- |
|  |  |  |  |
| 1. | Parity is accounted for | Yes | No |
| 2. | Planned home birth determined at onset of labour | Yes | No |
| 3. | Planned home births represent all home births during the study period OR clear description of cases removed | Yes | No |
| 4. | Planned hospital births are at low obstetric risk OR include only those that would be eligible to have a home birth | Yes | No |

| **Part D: Risk of Bias** | | | |
| --- | --- | --- | --- |
| 1. | **Definition of Outcomes**  Were the primary outcomes clearly defined? | Yes | No |
| 2. | **Confounding**  Were the groups matched on prognostic factors?  If yes, list variables matched on: | Yes | No |
| 3. | **Loss to follow-up**  Is missing data reported? | Yes | No |
| 4. | Was there greater than 10% missing data for any of the outcomes investigated? | Yes | No |
| 5. | If yes, which outcomes were missing greater than 10% of data? | | |

| **Part E: Study Setting – Integration of Midwifery/ Home Birth** | | | | | | | |
| --- | --- | --- | --- | --- | --- | --- | --- |
|  | | | | **Source** | | | |
|  |  |  |  | This publication | Author | Additional source (listed below) | |
| 1. | Country, region: | | |  |  |  | |
| 2. | What types of care providers attended home births? | | |  |  |  | |
| 3. | Were there care providers who **only** attended home births?  If yes, who? | Yes | No |  |  |  | |
| 4. | What types of care providers attended hospital births? | | |  |  |  | |
| 5. | Were there care providers who **only** attended hospital births?  If yes, who? | Yes | No |  |  |  | |
| 6. | Describe a typical situation in which a woman was transferred from a planned home birth to hospital, assuming it was not an emergency. | | | | | | |
|  | a. Could a home birth care provider easily have a woman transferred to hospital by ambulance? | Yes | No |  |  |  | |
|  | b. Did home birth care providers have admitting rights/ privileges at hospitals? | Yes | No |  |  |  | |
|  | c. Would the care provider who was providing care at home continue to care for the woman in hospital? | Yes | No |  |  |  | |
|  | d. Would home birth care providers and their clients be treated respectfully by other care providers while in hospital? | Yes | No |  |  |  | |
| 7a. | Were women required to pay a private fee to have a home birth at the time the study was carried out? | Yes | No |  |  |  | |
| 7b. | Were women required to pay a private fee to have a hospital birth at the time the study was carried out? | Yes | No |  |  |  | |
| 8. | Did the College of Obstetricians/ Gynecologists in the country have a statement about home birth at the time the study was carried out?  If yes: | Yes | No |  |  |  | |
|  | Does this statement support home birth? | Yes | No |  |  |  | |
|  | Does this statement not support but not oppose home birth? | Yes | No |  |  |  | |
|  | Does this statement oppose home birth? | Yes | No |  |  |  | |
|  | Were home birth care providers regulated at the time the study was carried out? | Yes | No |  |  |  | |
| 9. | Describe the training that home birth care providers received. | | |  |  |  | |
| 10. | Were there policy statements/regulations/ clinical practice guidelines that home birth care providers used to determine eligibility for home births at the time the study was carried out? | Yes | No |  |  |  | |
|  | If yes, what were the criteria that were used to determine eligibility for a home birth? | | |  |  |  | |
| 11. | Describe how information about eligibility and decisions about intended place of birth were recorded. | | | | | | |
|  | a. Who recorded the information? | | |  |  |  | |
|  | b. Where was the information recorded? | | |  |  |  | |
|  | c. Was there a specific form that was routinely used to collect this information in practice?  If yes, what items did this form collect? | Yes | No |  |  |  | |
|  | d. At what point(s) in pregnancy was this information collected? | | |  |  |  | |
| 12. | Would you consider this jurisdiction to be supportive of home birth based on the information provided in the manuscript, from the author and from other sources? | | | | | Yes | No |
| 13. | Provide references to additional sources of information and use numbers to indicate sources referred to above:  1.  2.  3.  4. | | | | | | |

| **Part F: Study Design and Quality** | | | | |
| --- | --- | --- | --- | --- |
| 1. | From what years were the data collected? |  | | |
| 2. | What was the source of data for the homebirth (intervention) group? |  | | |
| 3. | Was this prospective data collection?  Was this retrospective data collection? | | Yes  Yes | No  No |
| 4. | Are the eligibility criteria for the planned homebirth (intervention) group clearly defined? | | Yes | No |
| 5. | How was the outset of labour defined in the home birth group? | | | |
| 6. | Was there more than one comparison group? | | Yes | No |
|  | If yes, what were the names of the groups? | | | |
| **Complete an additional form for each hospital comparison group (Parts F to L).** | | | | |
| 7. | What is the name of the comparison group described here? |  | | |
| 8. | What was the source of data for the hospital birth (comparison) group? |  | | |
| 9. | Was this prospective data collection?  Was this retrospective data collection? | | Yes  Yes | No  No |
| 10. | Are the eligibility criteria for the planned hospital (comparison) group clearly defined? | | Yes | No |
| 11. | How was the outset of labour defined in the comparison group? | | | |

| **Part G: Participants/Intervention** | | |
| --- | --- | --- |
| 1. | Number in home birth group |  |
| 2. | Number in comparison group |  |
| 3. | Eligibility Criteria for homebirth (intervention) group |  |
| 4. | How was “low-risk” defined in the homebirth (intervention) group? |  |
| 5. | Eligibility Criteria for hospital birth (comparison) group |  |
| 6. | How was “low-risk” defined in the hospital (comparison) group? |  |

| **Part H: Baseline Characteristics** | | | | |
| --- | --- | --- | --- | --- |
|  | | | **Home Birth**  **(Intervention)** | **Hospital Birth (Comparison)** |
| **1.** | **Age** | | | |
|  | Categories: |  |  |  |
|  |  |  |  |  |
|  |  |  |  |  |
|  |  |  |  |  |
|  |  |  |  |  |
| **2.** | **Gestational Age** | | | |
|  | Categories: |  |  |  |
|  |  |  |  |  |
|  |  |  |  |  |
| **3.** | What was the prevalence of each method of determining gestational age in each group? | | | |
|  | Ultrasound | |  |  |
|  | Last menstrual period | |  |  |
|  | Not described | |  |  |
| **4.** | **Parity** | | | |
|  | Categories: | Para 0 |  |  |
|  |  | Para 1+ |  |  |
| **5.** | **Ethnic background** | | | |
|  | Categories: |  |  |  |
|  |  |  |  |  |
|  |  |  |  |  |
|  |  |  |  |  |
|  |  |  |  |  |
| **6.** | **Education** | | | |
|  | Categories: |  |  |  |
|  |  |  |  |  |
|  |  |  |  |  |
|  |  |  |  |  |
|  |  |  |  |  |
| **7.** | **Income** | | | |
|  | Categories: |  |  |  |
|  |  |  |  |  |
|  |  |  |  |  |
|  |  |  |  |  |
|  |  |  |  |  |
| **8.** | **Family structure/ marital status** | | | |
|  | Categories: |  |  |  |
|  |  |  |  |  |
|  |  |  |  |  |
|  |  |  |  |  |
|  |  |  |  |  |

| **Part I: Neonatal Outcomes** | | | | | | | | | | | | | | | | | | | | | | | | | | | |
| --- | --- | --- | --- | --- | --- | --- | --- | --- | --- | --- | --- | --- | --- | --- | --- | --- | --- | --- | --- | --- | --- | --- | --- | --- | --- | --- | --- |
| **1.** | **Infant mortality (excluding malformed infants)** | | | | | | | | | | | | | | Yes | | | | | | | | | No | | | |
| 2. | Definition or Categorisation: | | | | | | | | | | | | | | | | | | | | | | | | | | |
| 3. | Was parity accounted for? | | | | | | | | | | | | | | Yes | | | | | | | | | No | | | |
| 4. | If yes, what method was used to account for parity? | | | | | | | | | | | | | | | | | | | | | | | | | | |
| 5. |  | | | Experienced outcome | | | | | | | Did not experience outcome | | | | | | Total | | | | | | | | | | |
|  | Home Birth Group | | |  | | | | | | |  | | | | | |  | | | | | | | | | | |
|  | Comparison Group | | |  | | | | | | |  | | | | | |  | | | | | | | | | | |
|  | | | | | | | | | | | | | | | | | | | | | | | | | | | |
| 6. | Crude odds ratio: |  | | | | | | 95% CI: | | | | | |  | | | | | | | | | | | | | |
| 7. | Adjusted odds ratio: |  | | | | | | 95% CI: | | | | | |  | | | | | | | | | | | | | |
| 8. | Variables accounted for in adjusted odds ratio: | | | | | | | | | | | | | | | | | | | | | | | | | | |
|  | | | | | | | | | | | | | | | | | | | | | | | | | | | |
| **9.** | **Infant mortality (including malformed infants)** | | | | | | | | | | | | | | Yes | | | | | | | | | No | | | |
| 10. | Definition or Categorisation: | | | | | | | | | | | | | |  | | | | | | | | |  | | | |
| 11. | Was parity accounted for? | | | | | | | | | | | | | | Yes | | | | | | | | | No | | | |
| 12. | If yes, what method was used to account for parity? | | | | | | | | | | | | | | | | | | | | | | | | | | |
| 13. |  | | | | | Experienced outcome | | | | | Did not experience outcome | | | | | | Total | | | | | | | | | | |
|  | Home Birth Group | | | | |  | | | | |  | | | | | |  | | | | | | | | | | |
|  | Comparison Group | | | | |  | | | | |  | | | | | |  | | | | | | | | | | |
|  | | | | | | | | | | | | | | | | | | | | | | | | | | | |
| 14. | Crude odds ratio: | | | |  | | | | 95% CI: | | | | |  | | | | | | | | | | | | | |
| 15. | Adjusted odds ratio: | | | |  | | | | 95% CI: | | | | |  | | | | | | | | | | | | | |
| 16. | Variables accounted for in adjusted odds ratio: | | | | | | | | | | | | | | | | | | | | | | | | | | |
|  | | | | | | | | | | | | | | | | | | | | | | | | | | | |
| **17.** | **Neonatal Resuscitation** | | | | | | | | | | | | | | | | | | | | Yes | | | | | No | |
| 18. | Definition or Categorisation: | | | | | | | | | | | | | | | | | | | | | | | | | | |
| 19. | Was parity accounted for? | | | | | | | | | | | | | | | | | | | | Yes | | | | | No | |
| 20. | If yes, what method was used to account for parity? | | | | | | | | | | | | | | | | | | | | | | | | | | |
| 21. |  | Experienced outcome | | | | | | | | Did not experience outcome | | | | | | | | Total | | | | | | | | | |
|  | Home Birth Group |  | | | | | | | |  | | | | | | | |  | | | | | | | | | |
|  | Comparison Group |  | | | | | | | |  | | | | | | | |  | | | | | | | | | |
|  | | | | | | | | | | | | | | | | | | | | | | | | | | | |
| 22. | Crude odds ratio: |  | | | | | | | | 95% CI: | | | | | | | |  | | | | | | | | | |
| 23. | Adjusted odds ratio: |  | | | | | | | | 95% CI: | | | | | | | |  | | | | | | | | | |
| 24. | Variables accounted for in adjusted odds ratio: | | | | | | | | | | | | | | | | | | | | | | | | | | |
|  | | | | | | | | | | | | | | | | | | | | | | | | | | | |
| **25.** | **Apgar Score < 7 at 1 minute** | | | | | | | | | | | | | | | | | | | | | | Yes | | | | No |
| 26. | Definition or Categorisation: | | | | | | | | | | | | | | | | | | | | | | | | | | |
| 27. | Was parity accounted for? | | | | | | | | | | | | | | | | | | | | | | Yes | | | | No |
| 28. | If yes, what method was used to account for parity? | | | | | | | | | | | | | | | | | | | | | | | | | | |
| 29. |  | | | Experienced outcome | | | | | | | | Did not experience outcome | | | | Total | | | | | | | | | | | |
|  | Home Birth Group | | |  | | | | | | | |  | | | |  | | | | | | | | | | | |
|  | Comparison Group | | |  | | | | | | | |  | | | |  | | | | | | | | | | | |
|  | | | | | | | | | | | | | | | | | | | | | | | | | | | |
| 30. | Crude odds ratio: | | |  | | | | | | | | 95% CI: | | | |  | | | | | | | | | | | |
| 31. | Adjusted odds ratio: | | |  | | | | | | | | 95% CI: | | | |  | | | | | | | | | | | |
| 32. | Variables accounted for in adjusted odds ratio: | | | | | | | | | | | | | | | | | | | | | | | | | | |
|  | | | | | | | | | | | | | | | | | | | | | | | | | | | |
| **33.** | **Apgar Score < 7 at 5 minutes** | | | | | | | | | | | | | | | | | | | | Yes | | | | No | | |
| 34. | Definition or Categorisation: | | | | | | | | | | | | | | | | | | | | | | | | | | |
| 35. | Was parity accounted for? | | | | | | | | | | | | | | | | | | | | Yes | | | | No | | |
| 36. | If yes, what method was used to account for parity? | | | | | | | | | | | | | | | | | | | | | | | | | | |
| 37. |  | | | | | | Experienced outcome | | | | | | Did not experience outcome | | | | | | Total | | | | | | | | |
|  | Home Birth Group | | | | | |  | | | | | |  | | | | | |  | | | | | | | | |
|  | Comparison Group | | | | | |  | | | | | |  | | | | | |  | | | | | | | | |
|  | | | | | | | | | | | | | | | | | | | | | | | | | | | |
| 38. | Crude odds ratio: | | | | | |  | | | | | | 95% CI: | | | | | |  | | | | | | | | |
| 39. | Adjusted odds ratio: | | | | | |  | | | | | | 95% CI: | | | | | |  | | | | | | | | |
| 40. | Variables accounted for in adjusted odds ratio: | | | | | | | | | | | | | | | | | | | | | | | | | | |
|  | | | | | | | | | | | | | | | | | | | | | | | | | | | |
| **41.** | **Admission to Neonatal Intensive Care Unit (NICU)** | | | | | | | | | | | | | | | | | | | | | Yes | | | | | No |
| 42. | Definition or Categorisation: | | | | | | | | | | | | | | | | | | | | | | | | | | |
| 43. | Was parity accounted for? | | | | | | | | | | | | | | | | | | | | | Yes | | | | | No |
| 44. | If yes, what method was used to account for parity? | | | | | | | | | | | | | | | | | | | | | | | | | | |
| 45. |  | | Experienced outcome | | | | | | | | | | Did not experience outcome | | | | | | | Total | | | | | | | |
|  | Home Birth Group | |  | | | | | | | | | |  | | | | | | |  | | | | | | | |
|  | Comparison Group | |  | | | | | | | | | |  | | | | | | |  | | | | | | | |
|  | | | | | | | | | | | | | | | | | | | | | | | | | | | |
| 46. | Crude odds ratio: | |  | | | | | | | | | | 95% CI: | | | | | | |  | | | | | | | |
| 47. | Adjusted odds ratio: | |  | | | | | | | | | | 95% CI: | | | | | | |  | | | | | | | |
| 48. | Variables accounted for in adjusted odds ratio: | | | | | | | | | | | | | | | | | | | | | | | | | | |

| **Part J: Maternal outcomes** | | | | | | | | |
| --- | --- | --- | --- | --- | --- | --- | --- | --- |
| **1.** | **Maternal mortality** | | | | Yes | | | No |
| 2. | Definition or Categorisation: | | | | | | | |
| 3. | Was parity accounted for? | | | | Yes | | | No |
| 4. | If yes, what method was used to account for parity? | | | | | | | |
| 5. |  | Experienced outcome | Did not experience outcome | Total | | | | |
|  | Home Birth Group |  |  |  | | | | |
|  | Comparison Group |  |  |  | | | | |
|  | | | | | | | | |
| 6. | Crude odds ratio: |  | 95% CI: |  | | | | |
| 7. | Adjusted odds ratio: |  | 95% CI: |  | | | | |
| 8. | Variables accounted for in adjusted odds ratio: | | | | | | | |
|  | | | | | | | | |
| **9.** | **Postpartum hemorrhage** | | | | Yes | | | No |
| 10. | Definition or Categorisation: | | | | | | | |
| 11. | How was blood loss estimated at home births? | | | | | | | |
| 12. | How was blood loss estimated at hospital births? | | | | | | | |
| 13. | Was parity accounted for? | | | Yes | | No | | |
| 14. | If yes, what method was used to account for parity? | | | | | | | |
| 15. |  | Experienced outcome | Did not experience outcome | Total | | | | |
|  | Home Birth Group |  |  |  | | | | |
|  | Comparison Group |  |  |  | | | | |
|  | | | | | | | | |
| 16. | Crude odds ratio: |  | 95% CI: |  | | | | |
| 17. | Adjusted odds ratio: |  | 95% CI: |  | | | | |
| 18. | Variables accounted for in adjusted odds ratio: | | | | | | | |
|  | | | | | | | | |
| **19.** | **3^rd^ or 4^th^ degree perineal laceration** | | | | Yes | | | No |
| 20. | Definition or Categorisation: | | | | | | | |
| 21. | Was parity accounted for? | | | | Yes | | | No |
| 22. | If yes, what method was used to account for parity? | | | | | | | |
| 23. |  | Experienced outcome | Did not experience outcome | Total | | | | |
|  | Home Birth Group |  |  |  | | | | |
|  | Comparison Group |  |  |  | | | | |
|  | | | | | | | | |
| 24. | Crude odds ratio: |  | 95% CI: |  | | | | |
| 25. | Adjusted odds ratio: |  | 95% CI: |  | | | | |
| 26. | Variables accounted for in adjusted odds ratio: | | | | | | | |
|  | | | | | | | | |
| **27.** | **Infection** | | | | Yes | | | No |
| 28. | Definition or Categorisation: | | | | | | | |
| 29. | Was parity accounted for? | | | | Yes | | No | |
| 30. | If yes, what method was used to account for parity? | | | | | | | |
| 31. |  | Experienced outcome | Did not experience outcome | Total | | | | |
|  | Home Birth Group |  |  |  | | | | |
|  | Comparison Group |  |  |  | | | | |
|  | | | | | | | | |
| 32. | Crude odds ratio: |  | 95% CI: |  | | | | |
| 33. | Adjusted odds ratio: |  | 95% CI: |  | | | | |
| 34. | Variables accounted for in adjusted odds ratio: | | | | | | | |

| **Part K: Interventions** | | | | | | | | |
| --- | --- | --- | --- | --- | --- | --- | --- | --- |
| **1.** | **Oxytocin augmentation** | | | | Yes | | | No |
| 2. | Definition or Categorisation: | | | | | | | |
| 3. | Was parity accounted for? | | | | Yes | | | No |
| 4. | If yes, what method was used to account for parity? | | | | | | | |
| 5. |  | Experienced outcome | Did not experience outcome | Total | | | | |
|  | Home Birth Group |  |  |  | | | | |
|  | Comparison Group |  |  |  | | | | |
|  | | | | | | | | |
| 6. | Crude odds ratio: |  | 95% CI: |  | | | | |
| 7. | Adjusted odds ratio: |  | 95% CI: |  | | | | |
| 8. | Variables accounted for in adjusted odds ratio: | | | | | | | |
|  | | | | | | | | |
| **9.** | **Epidural anesthesia/analgesia** | | | | Yes | | | No |
| 10. | Definition or Categorisation: | | | | | | | |
| 11. | Was parity accounted for? | | | | Yes | | No | |
| 12. | If yes, what method was used to account for parity? | | | | | | | |
| 13. |  | Experienced outcome | Did not experience outcome | Total | | | | |
|  | Home Birth Group |  |  |  | | | | |
|  | Comparison Group |  |  |  | | | | |
|  | | | | | | | | |
| 14. | Crude odds ratio: |  | 95% CI: |  | | | | |
| 15. | Adjusted odds ratio: |  | 95% CI: |  | | | | |
| 16. | Variables accounted for in adjusted odds ratio: | | | | | | | |
|  | | | | | | | | |
| **17.** | **Episiotomy** | | | | Yes | | | No |
| 18. | Definition or Categorisation: | | | | | | | |
| 19. | Was parity accounted for? | | | | | Yes | | No |
| 20. | If yes, what method was used to account for parity? | | | | | | | |
| 21. |  | Experienced outcome | Did not experience outcome | Total | | | | |
|  | Home Birth Group |  |  |  | | | | |
|  | Comparison Group |  |  |  | | | | |
|  | | | | | | | | |
| 22. | Crude odds ratio: |  | 95% CI: |  | | | | |
| 23. | Adjusted odds ratio: |  | 95% CI: |  | | | | |
| 24. | Variables accounted for in adjusted odds ratio: | | | | | | | |
|  | | | | | | | | |
| **25.** | **Operative vaginal delivery (vacuum or forceps)** | | | | Yes | | | No |
| 26. | Definition or Categorisation: | | | | | | | |
| 27. | Was parity accounted for? | | | | Yes | | | No |
| 28. | If yes, what method was used to account for parity? | | | | | | | |
| 29. |  | Experienced outcome | Did not experience outcome | Total | | | | |
|  | Home Birth Group |  |  |  | | | | |
|  | Comparison Group |  |  |  | | | | |
|  | | | | | | | | |
| 30. | Crude odds ratio: |  | 95% CI: |  | | | | |
| 31. | Adjusted odds ratio: |  | 95% CI: |  | | | | |
| 32. | Variables accounted for in adjusted odds ratio: | | | | | | | |
|  | | | | | | | | |
| **33.** | **Caesarean section** | | | | Yes | | | No |
| 34. | Definition or Categorisation: | | | | | | | |
| 35. | Was parity accounted for? | | | | Yes | | No | |
| 36. | If yes, what method was used to account for parity? | | | | | | | |
| 37. |  | Experienced outcome | Did not experience outcome | Total | | | | |
|  | Home Birth Group |  |  |  | | | | |
|  | Comparison Group |  |  |  | | | | |
|  | | | | | | | | |
| 38. | Crude odds ratio: |  | 95% CI: |  | | | | |
| 39. | Adjusted odds ratio: |  | 95% CI: |  | | | | |
| 40. | Variables accounted for in adjusted odds ratio: | | | | | | | |

| **Part L: Transfer Rates** | | | | | | | |
| --- | --- | --- | --- | --- | --- | --- | --- |
| **1.** | **Actual place of birth reported** | | | | | Yes | No |
| 2. |  | Intended home birth group | | Intended hospital (comparison) group | | | |
|  | Total n |  | |  | | | |
|  | Actual place of birth: |  | |  | | | |
|  | Home |  | |  | | | |
|  | Hospital |  | |  | | | |
|  | Other: |  | |  | | | |
|  | Unknown |  | |  | | | |
| **3.** | **Emergency transfers** | | | | | Yes | No |
| 4. | Definition of **emergency** transfer in home birth group: | | | | | | |
| 5. | Definition of **emergency** transfer in comparison group: | | | | | | |
|  |  | | Intended home birth group | | Intended hospital (comparison) group | | |
|  | Total n | |  | |  | | |
| 6. | Transferred (to hospital from home or from hospital to another department or to another hospital) | |  | |  | | |
|  |  | |  | |  | | |
| 7. | **Emergency** transfer | |  | |  | | |
|  | Prior to birth | |  | |  | | |
|  | After birth, prior to delivery of placenta | |  | |  | | |
|  | After delivery of placenta | |  | |  | | |
|  | Not emergency | |  | |  | | |
|  | Prior to birth | |  | |  | | |
|  | After birth, prior to delivery of placenta | |  | |  | | |
|  | After delivery of placenta | |  | |  | | |

| **Part M: Miscellaneous** | | | | |
| --- | --- | --- | --- | --- |
| 1. | Funding source |  | | |
| 2. | Key conclusions |  | | |
| 3. | Miscellaneous comments from study authors |  | | |
| 4. | References to other relevant studies |  | | |
| 5. | Clarification required | | Yes | No |
|  | If yes, list items that require clarification: | | | |
