# Supplementary material for: Protocol: systematic review and meta-analyses of birth outcomes for women who intend at the onset of labour to give birth at home compared to women of low obstetrical risk who intend to give birth in hospital
Source: Syst Rev. 2014 May 29;3:55. doi: 10.1186/2046-4053-3-55 (PMC4046441; doi:10.1186/2046-4053-3-55)
Supplement: Additional file 4 — “Questionnaire for Authors of Included Studies”. Description: This file includes a questionnaire to be sent along with an accompanying cover letter to authors of all included studies to ascertain information about the integration of home birth into the health care system in the region at the time that each study was conducted. [file 2046-4053-3-55-S4.docx]

**Questionnaire for Authors of Included Studies**

**To be used as the body of an email:**

Dear [insert author’s name],

We are currently working on a meta-analysis to determine if women planning a home birth at the outset of labour are more or less likely to experience a foetal or neonatal loss compared to a similar cohort of low risk women planning hospital birth. We are including studies that used a control group that was comprised of women considered to be low risk who planned a hospital birth at the outset of labour. In addition the data analyses must have been based on the **planned** place of birth at the outset of labour rather than by the actual place of delivery.

Your publication meets our criteria and we are contacting you to request additional information so that we can further understand the setting from which the data were collected.

Attached you will find a short questionnaire that we are asking you to complete and return to us by email by [insert date] We hope to publish our findings in a peer-reviewed journal. The information that you provide may be included in our publication as part of a description of the included studies.

If you have any questions or require any clarification, please feel free to contact me by email (huttone@mcmaster.ca) or phone (905-525-9140 ext 26654).

Thank you very much.

Eileen Hutton, PhD

Please complete the following questions about home birth practices for the time period and the region from which your study data were collected. Once completed, please return to Eileen Hutton (huttone@mcmaster.ca).

1. What types of care providers attended planned **home** births?
   a. Were there care providers who **only** attended home births? If yes, who?
2. What types of care providers attended planned **hospital** births?
   a. Were there care providers who **only** attended hospital births? If yes, who?
3. Please describe a typical situation in which a woman was transferred from a planned home birth to hospital, assuming it was not an emergency.
   a. Could a home birth care provider easily have a woman transferred to hospital by ambulance?
   b. Did home birth care providers have admitting rights/ privileges at hospitals where the woman would be admitted?
   c. Would the care provider who was providing care at home continue to care for the woman in hospital?
   d. Would home birth care providers and their clients be treated respectfully by other care providers while in hospital?
4. Were women required to pay a private fee to have a home birth at the time the study was carried out?
5. Were women required to pay a private fee to have a hospital birth at the time the study was carried out?
6. Did the College of Obstetricians/Gynecologists in your country have a statement about home birth at the time the study was carried out?
   a. If yes, did the statement support home birth, oppose home birth or neither support nor oppose home birth?
   b. If yes, is a copy of the statement available?
7. Were home birth care providers regulated at the time the study was carried out?
8. Please describe the training that home birth care providers received.
9. Were there policy statements/regulations/ clinical practice guidelines that home birth care providers used to determine eligibility for home births at the time the study was carried out?
   a. If yes, can these be attached or can you describe the criteria that were used to determine eligibility for a home birth?
10. Please describe how information about eligibility and decisions about intended place of birth were recorded.
    a. Who recorded the information?
    b. Where was the information recorded?
    c. Was there a specific form that was routinely used to collect this information in practice? If yes, what items did this form collect?
    d. At what point(s) in pregnancy was this information collected?

In addition, we request clarification about the following aspects of your study:

**Note: These questions will be different for each author based on what we have identified as needing further clarification.**

11. To be determined for each study included.
